# Case report: Somatic mutations in microtubule dynamics-associated genes in patients with WNT-medulloblastoma tumors

**DOI:** 10.3389/fonc.2022.1085947

**Published:** 2023-01-12

**Authors:** Rostislav Skitchenko, Yulia Dinikina, Sergey Smirnov, Mikhail Krapivin, Anna Smirnova, Daria Morgacheva, Mykyta Artomov

**Affiliations:** ^1^ Almazov National Medical Research Centre, St. Petersburg, Russia; ^2^ Computer Technologies Laboratory, ITMO University, St. Petersburg, Russia; ^3^ The Institute for Genomic Medicine, Nationwide Children’s Hospital, Columbus, OH, United States; ^4^ Department of Pediatrics, Ohio State University, Columbus, OH, United States

**Keywords:** medulloblastoma, exome sequence data, somatic mutation analysis, Wnt, microtubule - associated proteins

## Abstract

Medulloblastoma (MB) is the most common pediatric brain tumor which accounts for about 20% of all pediatric brain tumors and 63% of intracranial embryonal tumors. MB is considered to arise from precursor cell populations present during an early brain development. Most cases (~70%) of MB occur at the age of 1–4 and 5–9, but are also infrequently found in adults. Total annual frequency of pediatric tumors is about 5 cases per 1 million children. WNT-subtype of MB is characterized by a high probability of remission, with a long-term survival rate of about 90%. However, in some rare cases there may be increased metastatic activity, which dramatically reduces the likelihood of a favorable outcome. Here we report two cases of MB with a histological pattern consistent with desmoplastic/nodular (DP) and classic MB, and genetically classified as WNT-MB. Both cases showed putative causal somatic protein truncating mutations identified in microtubule-associated genes: *ARID2*, *TUBB4A*, and *ANK3*.

## Introduction

Medulloblastoma (MB) – is a solid neuroepithelial tumor arising from the cerebellum. MB accounts for about 20% of all childhood brain tumors and 63% of intracranial embryonal tumors (1). MB is considered to arise from precursor cell populations present during an early brain development (2). Most cases (~70%) of MB occur at the age of 1–4 and 5–9, but are also found in adults (3). Total annual frequency of pediatric tumors is about 5 cases per 1 million children (1).

WHO declares two classifications of MB according to the method of diagnosis: histologically determined and genetically determined (4). Both groups are divided into several subgroups according to the immunohistochemical and genetic features, respectively (4). For histologically determined MB there are the following subgroups: 1) classic MB; 2) Desmoplastic/nodular MB; 3) MB with extensive nodularity; 4) Large cell/Anaplastic MB; 5) MB not otherwise specified (4). In turn, the following subgroups are distinguished for genetically defined MB: 1) WNT‐activated MB; 2) SHH‐activated, TP53‐wild‐type MB; 3) SHH‐activated, TP53‐mutant MB; 4) Non‐WNT/non‐SHH MB which is commonly divided into Group 3 and Group 4 MB (4).

Of all cases of MB, about 10% are of the wingless-type (WNT) (5). WNT-MB are usually located along the brain midline with involvement of the brainstem or cerebellar bundle and cerebellopontine angle cistern (6). WNT-MB is thought to arise from progenitor cells in the inferior rhombic lip of the developing brainstem. The vast majority of WNT tumors (~90%) contain a mutation affecting *CTNNB1*, which encodes β-catenin. Mutations in the tumor suppressor gene *APC* explain the majority of WNT-cases which do not have *CTNNB1* mutations (2).

Some studies suggested the existence of two subtypes of WNT: WNTα and WNTβ. The WNTα subtype occurs mainly in children and for 98% of cases is associated with chromosome 6 monosomy, whereas the WNTβ subtype occurs in older children and adults and infrequently (29%) has monosomy (7).

Here we present molecular diagnostics for two WNT-MB cases without chromosome 6 monosomy or mutations in *CTNNB1* and *APC*.

## Methods

### Clinical and genetic data collection

Patients were observed at Almazov National Medical Research Center in 2020-2022. Informed consent for molecular genetic testing was provided by parents of patients. The study was approved by the institutional ethics committee (Protocol #3502-22 from 21.02.2020).

Hematoxylin-eosin staining analysis was used for the purpose of histological classification of medulloblastomas.

A panel of three staining assays: 1) beta-catenin staining, 2) filamin A, 3) GAB1 was used to obtain immunohistochemical (IHC) confirmation of the diagnosis of MB and determine its genetically defined subtype. Ki-67 was assessed as a marker of proliferation activity along with synaptophysin expression, which is used to distinguish MB from embryonal tumor with multilayered rosettes (ETMR) and most atypical teratoid rhabdoid tumors (ATRT), which can potentially mimic MB (4).

Genomic DNA samples were prepared for sequencing using Kapa Biosystems (Roche) kits. To enrich the coding part of the genome, the TruSeq Exome Capture kit (Illumina) was used. The quality of the obtained libraries was controlled using the Fragment Analyzer. Sufficiency of the DNA quantity was assessed with the qPCR. After quality control and DNA quantity estimation, the pool of libraries was sequenced on 2 lanes of the Illumina NovaSeq 6000.

### Identification of putative causal variants

We assembled a list of 616 oncogenes, based on a broad list of 565 known oncogenes (8), and an overlapping set of 87 previously reported MB susceptibility genes (Sup. Materials – Susceptibility gene lists assembly; **Sup. Table S1**) (9–43).

Raw sequencing data in the form of FASTQ files were obtained using bcl2fastq v2.20 Conversion Software (Illumina). Germline and somatic variant calling were performed in accordance with GATK and Mutect2 best practices (44, 45).

Identified putative somatic variants were subjected to the quality filtration using the following thresholds based on GATK metrics: 1) DP>30, 2) GERMQ>90, 3) TLOD>3, 4) POPAF≥4, 5) ROQ>85.

We took extra caution in interpreting long indels. They often could be unreliably called and require a specialized approach for analysis (46, 47). Therefore, for indels greater than 10 nucleotides that could potentially be nominated as “causal” in both patients, we manually checked the alignment of the short reads with IGV. Such an approach was carried out consistently with common standards in the field (48).

All variant coordinates mentioned are based on the reference genome version of GRCh38 and are declared according to HGVS requirements (49). In assessing the functional effect of the variants found, we rely on the joint recommendations of Clinical Genome Resource (ClinGen), Cancer Genomics Consortium (CGC), and Variant Interpretation for Cancer Consortium (VICC) (Sup. Materials – Strategy for variant oncogenicity classification) (50).

To evaluate the functional importance of identified variants, we used databases of oncogenic variants. For this purpose, we used COSMIC (51) and PeCan (52) focused on pediatric oncology. Furthermore, we use PeCan’s built-in Pathogenicity Information Exchange (PIE) (53) tool, which estimates the pathogenicity of variants based on its sample cohort and additional estimates as Sorting Intolerant From Tolerant (SIFT) (54) score, likelihood ratio test (LRT) and Combined Annotation Dependent Depletion (CADD) (55) assessments.

## Results

### Report of cases

The patients were a female and a male of 10 years old (hereafter **Patient #1** and **Patient #2**) presented with complaints of headache, vomiting and visual impairments. Both patients underwent MRI analysis, surgical removal of the tumors, histological and immunohistochemical analysis. An exome sequencing from the blood and tumor DNA was performed and followed by germline and somatic variant calling. The sequencing data analysis was then performed to identify the likely genetic causes for the disease.

### Patient #1

**Figure 1:**
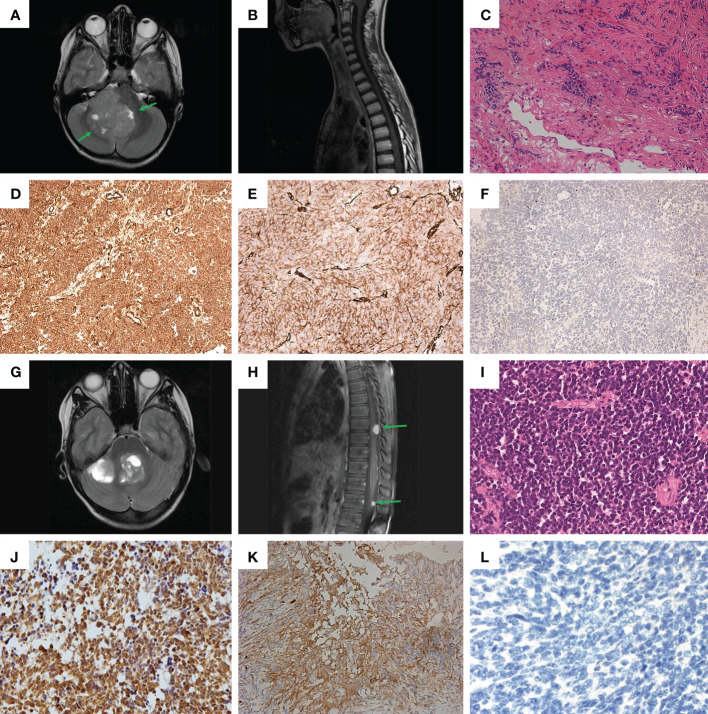
Clinical and histological characteristics. **(A, B)** – MRI screens in **Patient #1**: **(A)** brain; **(B)** spinal cord; **(C)** Hematoxylin-eosin staining of sample from **Patient #1**. **(D-F)** Immunohistochemical (IHC) staining of tumor sample from **Patient #1**: **(D)** beta-catenin; **(E)** filamin A; **(F)** GAB1. **(G, H)** MRI screens in **Patient #2**: **(G)** brain; **(H)** spinal cord. **(I)** Hematoxylin-eosin staining of sample from **Patient #2**. **(J–L)** IHC staining of tumor sample from **Patient #2**: **(J)** beta-catenin; **(K)** filamin A; **(L)** GAB1.

A multi-spiral CT scan (MSCT) of the brain revealed a formation in the cerebellum and brainstem as well as triventricular hydrocephalus and periventricular oedema. A magnetic resonance imaging (MRI) of the brain confirmed the results of the MSCT and additionally revealed a mass in the IV ventricle of the brain (**Figure 1A**); MRI screen of the spinal cord showed no signs of metastasis (**Figure 1B**). An additional optometric exam revealed signs of optic disc stasis. The patient was prescribed dexamethasone, which had a positive effect on reducing the headaches.

After 17 days of observation, a suboccipital bone-plastic craniotomy was performed under neurophysiological monitoring, with microsurgical removal of tumors of the cerebellum, IV ventricle and brainstem.

Further histological examination of the tumor fragments showed a highly cellular tissue sample of small cells with polymorphic hyperchromatic nuclei, with poor eosinophilic cytoplasm. Areas of nodular structure of light-colored cells were also present. The formation of Homer-Wright-type rosettes was noted. The sample was characterized by an increased number of mitoses, including atypical ones, and endothelial proliferation (**Figure 1C**). As a result, the tumor from **Patient #1** was assigned to the desmoplastic/nodular type of MB according to the WHO classification (4).

**Figure 2:**
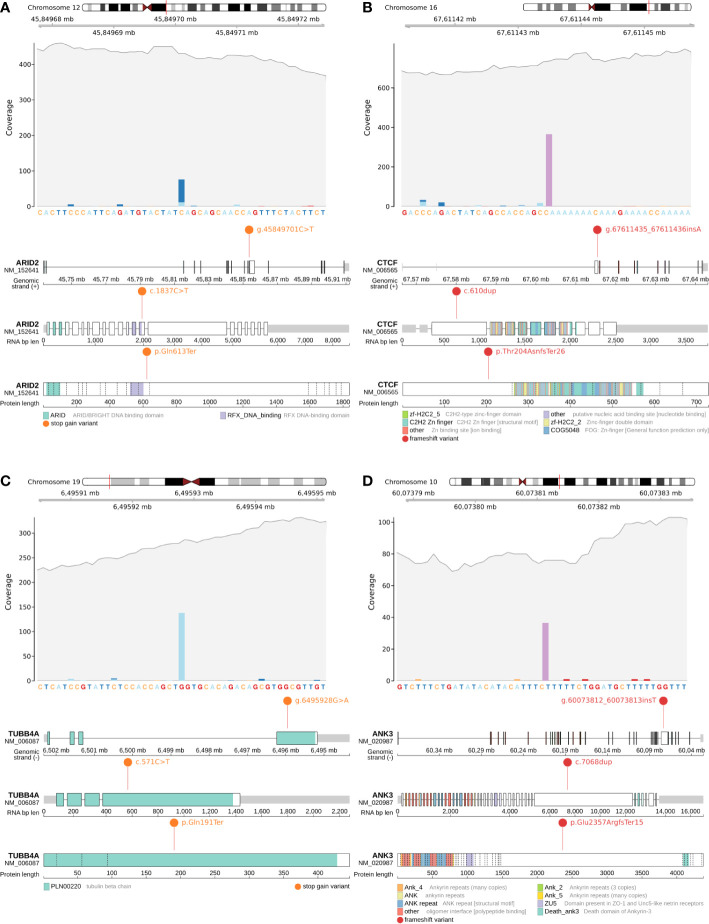
Candidate somatic protein truncating variants (GRCh38): **(A, B)** – coverage and functional effects of PTVs on DNA, RNA and protein level in Patient #1. **(A)**NC_000012.12:g.45849701C>T in *ARID2* (NM_152641:p.Gln613Ter); **(B)** NC_000016.10:g.67611435_67611436insA in *CTCF* (NM_006565:p.Thr204AsnfsTer26); **(C, D) ** – coverage and functional effects of PTVs on DNA, RNA and protein level in Patient #2. **(C)** NC_000019.10:g.6495928G>A in *TUBB4A* (NM_006087:p.Gln191Ter); **(D)** NC_000010.11:g.60073812_60073813insT in *ANK3* (NM_020987:p.Glu2357ArgfsTer15).

Immunohistochemical (IHC) analysis for the sample obtained from **Patient #1** revealed: 1) positive membrane-cytoplasmic and nuclear beta-catenin staining (**Figure 1D**); 2) positive cytoplasmic filamin A staining (**Figure 1E**); 3) negative GAB1 staining (**Figure 1F**). Therefore, the tumor was assigned to the WNT subtype, according to the genetically defined WHO classification (ICD-10-CM:C71.8; G97.9). Additionally, the proliferative activity of Ki-67 was assessed, which was about 25-30%, as well as synaptophysin expression (**Figure S1A**), which distinguished MB from ETMR and most ATRT, which can potentially mimic MB (4).

### Patient #2

MRI of the brain showed formation in the IV ventricle and right hemisphere of the cerebellum and internal hydrocephalus (**Figure 1G**). In addition, MRI of the spinal cord showed signs of spinal metastasis (**Figure 1H**).

After 5 days, a partial surgical removal of a tumor of the right cerebellar hemisphere, IV ventricle, was performed.

Histological examination revealed a monotonous, dense-, small- and blue-cellular malignant tumor with rosettes and little stroma and numerous mitoses (**Figure 1I**). As a result, in the course of histological examination, the preparation from **Patient #2** was assigned the classical type of MB according to the WHO classification (4).

**Patient #2** had the same set of IHC confirmations as **Patient #1**: 1) positive membrane-cytoplasmic and nuclear beta-catenin staining (**Figure 1J**); 2) positive cytoplasmic filamin A staining (**Figure 1K**); 3) negative GAB1 staining (**Figure 1L**). Thus, the results of IHC analysis suggest that the tumor should be assigned to the WNT subtype, according to the genetically defined WHO classification (ICD-10-CM: C71.8; G91.1, G96.8, G83.2). Additional IHC analysis yielded the following: 1) positive expression of synaptophysin (**Figure S1B**); 2) Proliferative activity Ki-67 on level 20-30%.

### Molecular diagnosis

Somatic variant calls were subjected to quality filtration to ensure only high-confidence somatic mutations entered the analysis (**Methods**). The chromosome 6 monosomy was ruled out for both patients using heterozygosity analysis that indicated presence of the two copies of the chromosome 6 (**Figure S2**). In total there were 50 and 37 good quality somatic variants for analysis in **Patient #1** and **Patient #2** respectively (**Sup. Tables S2, 3**). Out of these variants, 26 and 17 were eliminated from the analysis as non-coding, 3 and 1 as inframe indels, 2 and 1 were eliminated as synonymous for **Patient #1** and **Patient #2**, respectively. Furthermore, 9 and 11 variants each with ambiguous or missing annotation were excluded from the analysis for **Patient #1** and **Patient #2**, respectively.

Initially, we focused our analysis on missense variants and protein truncating variants (PTV). In the data, there were five and two missense variants and five PTV for each **Patient #1** and **Patient #2**, respectively.

**Patient #1** had only one variant in a gene from the list of MB susceptibility genes (87 genes list). For **Patient #2**, the genes from the MB susceptibility gene list did not contain any mutations.

None of the identified somatic missense variants was found in the two examined gene sets in both patients. Six of seven missense variants outside the lists of known oncogenes were eliminated as unlikely to affect any important conservative parts of the gene, as their missense deleteriousness (MPC) (56) score was ≤2 (**Sup. Table S4**).

**Patient #1** had only one mutation in a known oncogenic gene from the analyzed list – a stop gain somatic mutation (NC_000012.12:g.45849701C>T, NM_152641:p.Gln613Ter) in *ARID2* (**Figure 2A**), which disrupts cell cycle regulation and has previously been identified as a MB risk gene (87 genes list) (10, 11). Variant was found to be in a close proximity to RFX DNA-binding protein domain (the domain boundary is at amino acid 601). For **Patient #1** it was the only PTV within the MB susceptibility gene list (87 genes list) and/or expanded gene list (616 genes list).

Four other PTVs found in **Patient #1** were located in *NOBOX*, *SRRM2*, *CTCF*, *RAB11FIP4*. Upon screening of these variants in IGV (57), frameshifts in *SRRM2* and *RAB11FIP4* were eliminated because of the poor mapping quality (**Methods – Identification of putative causal variants**). Frameshift variant in *NOBOX* was excluded from consideration because of its specific expression only in testis and ovarian tissues as was indicated by GTEX (58) (**Sup. Table S5**).

*CTCF* is an evolutionarily conserved gene responsible for the spatial properties of chromatin, including its accessibility to chromatin, so the frameshift indel (NC_000016.10:g.67611435_67611436insA, NM_006565:p.Thr204AsnfsTer26) (**Figure 2B**) in *CTCF* can potentially be considered as a secondary priority cause of MB in **Patient #1**.

For **Patient #2**, none of the variants were found in the 87 genes list. Next, we considered an extended list of 616 oncogenes in which the long frameshift in *MAP2K4* was detected. We performed visual control of this PTV with IGV and eliminated this candidate variant due to poor mapping quality. In a further analysis, we considered variants in all genes and found 4 PTVs in *AP003062.1*, *KLHL4*, *ANK3, TUBB4A*. After visually screening all 4 variants in IGV (57), we discarded 2 long frameshifts in *AP003062.1* and *KLHL4* due to poor mapping quality (Methods – Identification of putative causal variants; **Sup. Table S6**).

The remaining pair of PTVs were stop gain somatic mutation (NC_000019.10:g.6495928G>A, NM_006087:p.Gln191Ter) in *TUBB4A* (rs1376427129, gnomAD_AF=6.57x10^-6^) (**Figure 2C**) and frameshift indel (NC_000010.11:g.60073812_60073813insT, NM_020987:p.Glu2357ArgfsTer15) in *ANK3* (**Figure 2D**).

Conclusively, taking into account clinical symptoms, IHC and genetic analyses the diagnosis was defined as WNT-β medulloblastomas without chromosome 6 monosomy and no known mutations in *CTNNB1* and *APC*. Novel identified risk variants align well with the previous knowledge of *ANK3*, *TUBB4A*, *ARID2* and *CTCF* functionality in cancer but the specific variants that were identified in these patients have not been observed previously. In addition, the role of these variants in pediatric tumors of the central nervous system has not been previously reported.

## Discussion

### Patient #1

The *ARID2* is a highly conservative gene (pLI=1) involved in various biological processes, including the cell cycle control, regulation of transcription and modification of chromatin structure and is a known tumor suppressor gene (8). The *ARID2* gene product functions as a subunit of the PBAF (SWI/SNF-B) chromatin remodeling complex, which promotes ligand-dependent transcriptional activation by nuclear receptors. It was previously known that *ARID2* co-immunoprecipitates with α-tubulin and that *ARID2* localizes to the spindle pole during mitosis (59). Rare somatic mutations in *ARID2* can lead to severe phenotypes, including MB. For one-third of WNT-MB cases, functional annotation of the recurrently altered genes revealed somatic dysregulation of chromatin modeling genes of the SWI/SNF family, which also includes *ARID2* (10, 60). PeCan (52) did not show an exact match for the p.Gln613Ter in *ARID2* in pediatric oncology reports. However, PeCan’s (52) built-in PIE classified p.Gln613Ter as “GOLD” [“truncation in gold gene (tumor suppressor)”], likewise based on LRT (“Deleterious”) and CADD (CADD=38, CADD_raw_=11.70) estimates. According to COSMIC, p.Gln613Ter in *ARID2*, has been reported several times in the database as a variant found in various cancer types, though not in the central nervous system (61–63). We categorize g.45849701C>T as “oncogenic” according to accumulated evidence, as suggested by Horak et al. (Sup. Materials – Strategy of variant oncogenicity classification) (50).

Considering *CTCF* as a secondary finding in **Patient 1** it is worth noting its properties of regulating chromatin spatial regulation. It is known that CTCF-binding sites often define topological associating chromatin domains (TAD) boundaries and removal of these sites can lead to a moderate upregulation of a nearby gene. Therefore, alterations in *CTCF* genotype may potentially lead to significant gene expression alterations (64–66). Variant p.Thr204AsnfsTer26 was found to have an exact match with ClinVar and was assessed as “pathogenic” (Variation ID: 280869). PeCan (52) has shown that variant p.Thr204AsnfsTer26 has already been reported several times in pediatric oncology studies of lymphoblastic leukemia and solid tumors (67–69). PIE classified p.Thr204AsnfsTer26 as “GOLD”. Additionally, COSMIC shows multiple lines of evidence in studies involving various tumor types (65, 70, 71). The abundance of evidence in the database allows this variant to be identified as a cancer hotspot. *CTCF* is a very conservative gene, with almost no PTVs observed in germline DNA in large population-based cohorts (pLI=1), yet, there was no specific linkage to pediatric brain tumors reported to date. The accumulated evidence for g.67611435_67611436insA indicates that this is an “oncogenic” variant (Sup. Materials – Strategy of variant oncogenicity classification) (50).

### Patient #2

In a previous survival analysis study, *TUBB4A* expression in tumors was found to be associated with MB patients survival, suggesting that *TUBB4A* may have oncogenic properties (72). Interestingly, observed PTV is found in the last exon of the gene. Previous studies indicated that in other genes, including cancer genes, such mutations result in gain-of-function effect (73–75). This is consistent with the observation of lower expression of *TUBB4A* benefiting the survival. *TUBB4A* is non-conservative gene (pLI=0.11), which could potentially reduce the effect of PTV on viability. Missense mutations in *TUBB4A* are known to affect various neurological phenotypes, including those associated with cerebellar atrophy, early infantile encephalopathy, which may be due to the selective effects of different mutations on cells and microtubule dynamics (76).

Microtubules are components of the cytoskeleton that contribute to the morphology of axons and dendrites in neurons and facilitate the transport of cell cargos. In dividing cells, microtubules of polymerized α-/β-tubulin dimers control the process of mitosis at different stages of its course, which has been previously well studied (77, 78). Microtubules are prone to constant phases of polymerization and depolymerization, and changes in microtubule dynamics can lead to errors in chromosome segregation and chromosome instability, a key feature of oncological cells (78–81).

In cancer cells, changes in microtubules dynamics, often associated with cancer-specific tubulin isotypes and tubulin post-translational modifications, are involved in metastatic cell migration, drug resistance, and tumor vascularization (81, 82). It is important to clarify that the hyperfunction of tubulin motility in mitosis is also a molecular target for numerous “antitubulin agents”, which have been shown to interact with multiple sites on α- or β-tubulin and have been successfully used as chemotherapeutic agents to induce mitotic arrest and cancer cell death (83, 84).

The *ANK3* regulates the mitogen−activated protein kinase (MAPK) pathway related to extracellular matrix organization, cell motility through PTK2 signaling and somatodendritic inhibitory synapses, which determines its high conservativity (pLI=1) (85). Abnormalities in MAPK signaling are known to be associated with the process of metastasis and have long been proposed as targets for selective therapy for oncologies, since the presence or absence of metastasis often determines the prognosis of survival (85). But, even more importantly, that brain-specific *Ank3* is linked to microtubule dynamics through a GSK3/CRMP2-dependent mechanism, which has been confirmed using mouse models (86). There is evidence that increased *ANK3* expression in cancer tissues correlates with better survival in prostate cancer, suggesting that *ANK3* is a tumor suppressor gene (87).

Early gene expression studies in the hippocampus of Ank3+/- and Ank3+/+ mice revealed altered expression of 282 genes that were enriched with microtubule-related functions (86). *ANK3* binds microtubules directly or through the binding of microtubule-associated proteins at the plus-end stabilization cap, which prevents depolymerization and directly affects microtubule dynamics (88–90).

COSMIC and PeCan did not show an exact match with the p.Gln191Ter in *TUBB4A* and p.Glu2357ArgfsTer15 in *ANK3*, which makes it impossible to classify them as cancer hotspots. PIE has added evidence of p.Gln191Ter in *TUBB4A* oncogenicity through SIFT (“Damaging”), CADD (CADD=36, CADD_raw_=10.41) and LRT (“Deleterious”). PIE did not have sufficient information about p.Glu2357ArgfsTer15 in *ANK3*. Given involvement of these variants in oncological processes, the severity of the functional effect on the protein, and the available data from the survival analysis incline us to classify g.6495928G>A in *TUBB4A* as “variant of uncertain significance” and g.60073812_60073813insT in *ANK3* as “oncogenic” (Sup. Materials – Strategy of variant oncogenicity classification) (50).

Conclusively, we identified four candidate somatic mutations potentially explaining the MB onset in two pediatric patients and providing new biological insights into the mechanisms of the pediatric tumor development.

## Data availability statement

A full list of somatic mutations passing quality filtration is available in **Sup. Tables S1** and **S2**. Patients’ consents for clinical DNA sequencing do not permit free data sharing, however, reasonable requests for specific details of genotypes, not conflicting with consent could be accommodated by contacting the corresponding author.

## Ethics statement

The studies involving human participants were reviewed and approved by Almazov National Medical Research Center institutional ethics committee (Protocol # 3502-22 of 21.02.2020). Written informed consent to participate in this study was provided by the participants’ legal guardian/next of kin. Written informed consent was obtained from the minor(s)’ legal guardian/next of kin for the publication of any potentially identifiable images or data included in this article.

## Author contributions

RK, YD, MA designed the study. YD, SS, MK, AS, DM directly supervised patients and obtained biospecimen. RK, MA, analyzed the data. RK, MA wrote the manuscript. All authors contributed to the article and approved the submitted version.
