# Supplementary material for: Case report: Somatic mutations in microtubule dynamics-associated genes in patients with WNT-medulloblastoma tumors
Source: Front Oncol. 2023 Jan 12;12:1085947. doi: 10.3389/fonc.2022.1085947 (PMC9877404; doi:10.3389/fonc.2022.1085947)
Supplement: Supplementary file 1 [file DataSheet_1.pdf]

Somatic mutations in microtubule dynamics-associated genes in patients with WNT-medulloblastoma tumors

Supplementary Materials

Rostislav Skitchenko[1,2], Yulia Dinikina[1], Sergey Smirnov[1], Mikhail Krapivin[1], Anna Smirnova[1], Daria Morgacheva[1], Mykyta Artomov[1-4]

[1] - Almazov National Medical Research Centre, St. Petersburg, Russia
[2] - ITMO University, St. Petersburg, Russia
[3] - The Institute for Genomic Medicine, Nationwide Children's Hospital, Columbus, OH, USA
[4] - Department of Pediatrics, Ohio State University, Columbus, OH, USA

Correspondence: mykyta.artomov@nationwidechildrens.org

The authors declare no conflict of interest

Tables of content

Susceptibility gene lists assembly **2**

Strategy for variant oncogenicity classification **3**

Figures section **5**

References **7**

Susceptibility gene lists assembly

The composition of the oncogenes list was conducted through analyzing the publication by y Zhang, J. et al [1], which provided an extensive list of 565 oncogenes. This list of genes included five groups: 1) autosomal dominant cancer-predisposition genes (60), 2) autosomal recessive cancer-predisposition genes (29), 3) tyrosine kinase genes (23), 4) tumor-suppressor genes (58), 5) other cancer genes (395).

Medulloblastoma risk gene list was assembled through an extensive manual screening of more than 50 publications and its cross-links in pubmed and google scholar using various queries from combinations of search words: "medulloblastoma", "pediatric", "oncology", "neurooncology", "cancer", "genetics", "genes", "pathway", "landscape", "GWAS", "association". This resulted in a list of 87 confidently reported causal/associated genes (**Sup. Table 3**) from 35 peer-reviewed literature sources that may be directly or indirectly related to the pathogenesis of medulloblastoma.

Strategy for variant oncogenicity classification

In assessing the functional effect of the variants found, we rely on the joint recommendations of Clinical Genome Resource (ClinGen), Cancer Genomics Consortium (CGC), and Variant Interpretation for Cancer Consortium (VICC) [2]. Recommendations for classifying variants involve a process of assessing the nature of gene, functional effect of a variant, its population frequency, and determining additional characteristics such as conservativity. The scoring system is a 10-point scale, where points are awarded for satisfying the condition of the oncological variant. Horak et al propose 5 groups of variants according to increasing oncogenicity: 1) benign (score≤-7), 2) likely benign (-6≤score≤-1), 3) variant of uncertain significance (0≤score≤5), 4) likely oncogenic (6≤score≤9), 5) oncogenic (score≥10). All the information obtained was evaluated by a sum of points, on the basis of which a particular variant class is assigned.

To determine the oncogenicity class of a variant, first, the extent to which the gene can fit into the tumor suppressor group was assessed on the basis of peer-reviewed literature. The functional effect of the variant on the protein was then assessed. In the case where PTV is affecting tumor suppressor gene this gives 8 points to oncogenicity score, which at least defines the variant as “likely pathogenic”. After that, to make up for the missing two points the occurrence of the variant in other pediatric cancer studies is determined using PeCan[3,4]. Depending on hotspot frequency, 1, 2 or 4 points are added to the oncogenicity score. An additional source of points is to check the variant for its absence in the gnomAD population data, and the conservativity of the gene are determined by the integral pLI score. In case the variant is missing in gnomAD and the gene is conservative (pLI=1), 1 point is added for each condition. Missense badness, PolyPhen-2, and constraint (MPC) score is additionally checked for missense variants [5]. Points are deducted for the high frequency of the variant in gnomAD, for the silence effect of the variants, and for previously published evidence of a “benigness” of variant. Furthermore, we take into account computational evidence such as survival studies or CADD/SIFT/LRT scores, for which we could get a 1 point.

Variant oncogenicity for the identified putative causal variants:

1) NC_000012.12:g.45849701C>T in *ARID2* (sum=12):
    - It is a PTV in tumor suppressor gene – 8 points;
    - It is a cancer hotspot with low rate – 1 point;
    - It is absence in gnomAD – 1 point;
    - “High” estimates of LRT/CADD – 1 point;
    - It is a conservative gene (pLI) – 1 point.
2) NC_000016.10:g.67611435_67611436insA in *CTCF* (sum=16):
    - It is a PTV in tumor suppressor gene – 8 points;
    - It is a cancer hotspot with high rate – 4 points;
    - Exact match with ClinVar – 2 points;
    - It is absence in gnomAD – 1 point;
    - It is a conservative gene (pLI) – 1 point.
3) NC_000019.10:g.6495928G>A in *TUBB4A* (sum=2):
    - It is a PTV in oncogene (not a tumor suppressor gene) – 0 points;
    - It is not a cancer hotspot – 0 points;
    - It is ultra rare variant in gnomAD – 0 points;
    - It is not a conservativity gene (pLI=0.11) – 0 point;
    - “High” SIFT/LTR/CADD scores – 1 point;
    - There is survival analysis evidence – 1 point.
4) NC_000010.11:g.60073812_60073813insT in *ANK3* (sum=10):
    - It is a PTV in tumor suppressor gene – 8 points;
    - It is not a cancer hotspot – 0 points;
    - It is absence in gnomAD – 1 point;
    - It is a conservative gene (pLI) – 1 point.

Figures section

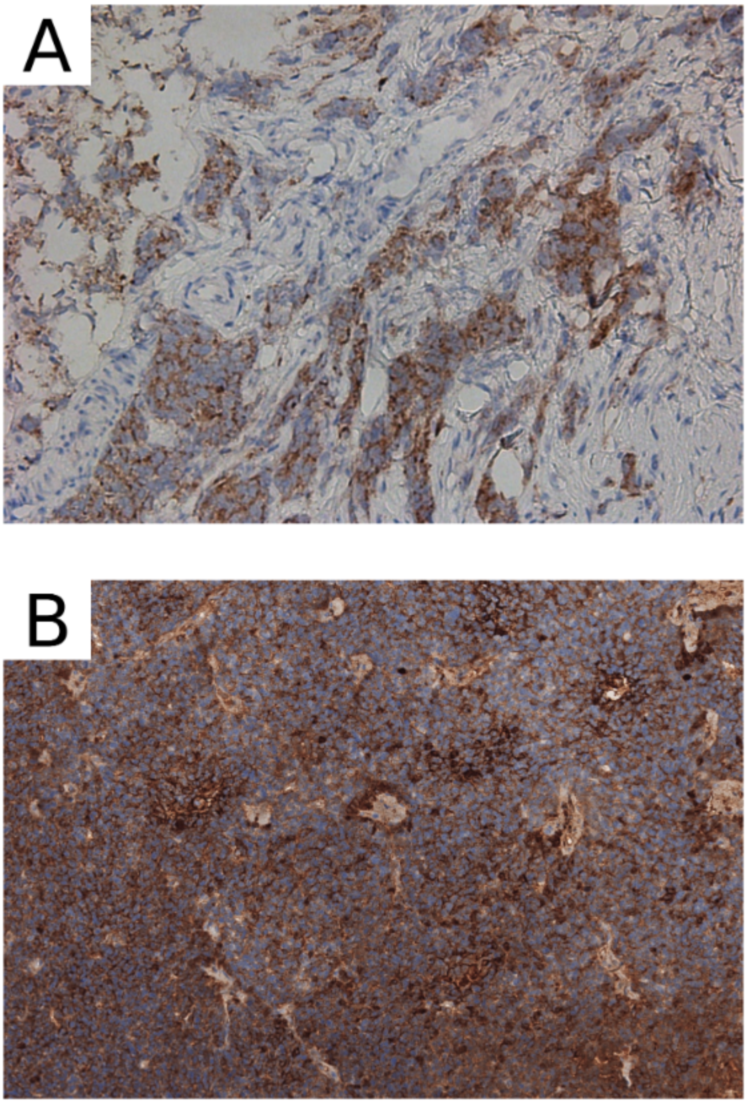

Figure S1. Synaptophysin staining for **Patient #1** (**A**) and **Patient #2** (**B**)

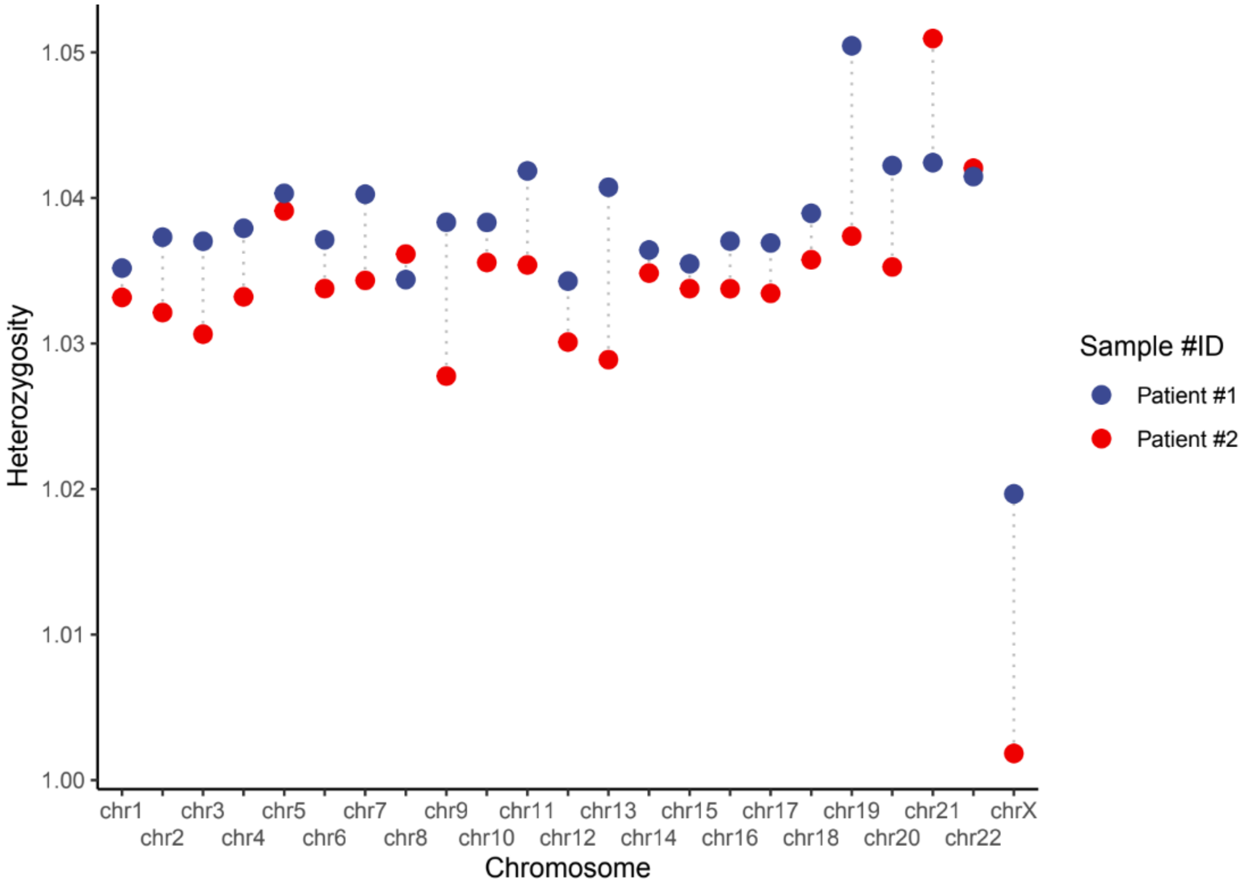

Figure S2. Per chromosome heterozygosity level for **Patient #1** and **Patient #2**

References

1. Zhang, J., Walsh, M.F., Wu, G., Edmonson, M.N., Gruber, T.A., Easton, J., Hedges, D., Ma, X., Zhou, X., Yergeau, D.A., et al. (2015). Germline mutations in predisposition genes in pediatric cancer. N. Engl. J. Med. *373*, 2336–2346.

2. Horak, P., Griffith, M., Danos, A.M., Pitel, B.A., Madhavan, S., Liu, X., Chow, C., Williams, H., Carmody, L., Barrow-Laing, L., et al. (2022). Standards for the classification of pathogenicity of somatic variants in cancer (oncogenicity): Joint recommendations of Clinical Genome Resource (ClinGen), Cancer Genomics Consortium (CGC), and Variant Interpretation for Cancer Consortium (VICC). Genet. Med. *24*, 986–998.

3. McLeod, C., Gout, A.M., Zhou, X., Thrasher, A., Rahbarinia, D., Brady, S.W., Macias, M., Birch, K., Finkelstein, D., Sunny, J., et al. (2021). St. Jude Cloud: A Pediatric Cancer Genomic Data-Sharing Ecosystem. Cancer Discov. *11*, 1082–1099.

4. Edmonson, M.N., Patel, A.N., Hedges, D.J., Wang, Z., Rampersaud, E., Kesserwan, C.A., Zhou, X., Liu, Y., Newman, S., Rusch, M.C., et al. (2019). Pediatric Cancer Variant Pathogenicity Information Exchange (PeCanPIE): a cloud-based platform for curating and classifying germline variants. Genome Res. *29*, 1555–1565.

5. Samocha, K.E., Kosmicki, J.A., Karczewski, K.J., O'Donnell-Luria, A.H., Pierce-Hoffman, E., MacArthur, D.G., Neale, B.M., and Daly, M.J. (2017). Regional missense constraint improves variant deleteriousness prediction. BioRxiv.
